# The microbiota structure in the cecum of laying hens contributes to dissimilar H_2_S production

**DOI:** 10.1186/s12864-019-6115-1

**Published:** 2019-10-23

**Authors:** Chun-Bo Huang, Lei Xiao, Si-Cheng Xing, Jing-Yuan Chen, Yi-Wen Yang, Yang Zhou, Wei Chen, Juan-Boo Liang, Jian-Dui Mi, Yan Wang, Yin-Bao Wu, Xin-Di Liao

**Affiliations:** 1 College of Animal Science, South China Agricultural University, Guangzhou, China; 2 Institute of Tropical Agriculture, University of Putra Malaysia, Serdang, Malaysia; 3 Ministry of Agriculture Key Laboratory of Tropical Agricultural Environment, South China Agricultural University, Guangzhou, China

**Keywords:** Odor, Cecum, Microbiota, Laying hen, Breed

## Abstract

**Background:**

Host genotype plays a crucial role in microbial composition of laying hens, which may lead to dissimilar odor gas production. The objective of this study was to investigate the relationship among layer breed, microbial structure and odor production.

**Results:**

Thirty Hy-Line Gray and thirty Lohmann Pink laying hens were used in this study to determine the impact of cecal microbial structure on odor production of laying hens. The hens were managed under the same husbandry and dietary regimes. Results of in vivo experiments showed a lower hydrogen sulfide (H_2_S) production from Hy-Line hens and a lower concentration of soluble sulfide (S^2−^) but a higher concentration of butyrate in the cecal content of the Hy-Line hens compared to Lohmann Pink hens (*P <* 0.05), which was consistent with the in vitro experiments (*P <* 0.05). However, ammonia (NH_3_) production was not different between genotypes (*P >* 0.05). Significant microbial structural differences existed between the two breed groups. The relative abundance of some butyrate producers (including *Butyricicoccus*, *Butyricimonas* and *Roseburia*) and sulfate-reducing bacteria (including *Mailhella* and *Lawsonia*) were found to be significantly correlated with odor production and were shown to be different in the 16S rRNA and PCR data between two breed groups. Furthermore, some bacterial metabolism pathways associated with energy extraction and carbohydrate utilization (oxidative phosphorylation, pyruvate metabolism, energy metabolism, two component system and secretion system) were overrepresented in the Hy-Line hens, while several amino acid metabolism-associated pathways (amino acid related enzymes, arginine and proline metabolism, and alanine-aspartate and glutamate metabolism) were more prevalent in the Lohmann hens.

**Conclusion:**

The results of this study suggest that genotype of laying hens influence cecal microbiota, which in turn modulates their odor production. Our study provides references for breeding and enteric manipulation for defined microbiota to reduce odor gas emission.

## Background

The poultry industry is a vital livestock sector producing meat and eggs for human consumption. The short digestive tract and fast chyme transit time (less than 2.5 h) in the upper intestine of chicken results in incomplete digestion and absorption of feed nutrients in the foregut [1, 2]. However, large quantities and diverse microorganisms inhabit in the cecum of laying hens, which prolongs the retention time of digesta to approximately 12–20 h. This latter process is known as microbial-based metabolism [3–5]. Previous studies have demonstrated that this “microbial organ” expands the biochemical potential in feed degradation, micronutrient production, odor emission, pathogen exclusion and immune system development in poultry [6–8].

It is well known that the ecological environment in the cecum of laying hens is composed of microorganisms and metabolites. Furthermore, it is generally accepted that the proportions and concentrations of these metabolites depend on the microbial composition, which to some degree, could be modulated by the composition and structure of the diet [9]. The degradation of undigested feed components (mainly undigested carbohydrates and proteins) and uric acid produce various bacterial metabolites. The rapid hydrolysis of uric acid and urea by microbial enzymes leads to the formation of ammonium, which is subsequently converted into NH_3_ and discharged into the atmosphere. The microbiota in the cecum of laying hens also contributes to sulfur metabolism, and the dissimilatory sulfate-reduction activity by sulfate-reducing bacteria (SRB) is the primary source for H_2_S formation and emission [10, 11]. Furthermore, the fermentation of undigested protein also contributes to NH_3_ and H_2_S discharge, but this pathway is not the dominant way for odor production [12–14]. In contrast, the fermentation of undigested carbohydrates by acid-producing bacteria provides numerous volatile fatty acids (VFAs), such as acetate, propionate and butyrate [15]. Previous studies found that an active carbohydrate fermentation (adequate production of VFAs) by gut acid-producing bacteria could result in the suppression of protein fermentation and partly inhibit the production of odor gas [16, 17].

At present, China has the largest number of laying hens in the world, with Lohmann and Hy-Line being the major breeds. Thus, there is increasing concern from the general public regarding odor emission from intensive poultry production. Long term exposure to odor gas reduces the productivity of animals and also induces diseases [18]. NH_3_ comprises the largest proportion of odor gas in livestock and poultry facilities [19]. In addition, H_2_S is also the predominant ones because the concentration of it only second to that of ammonia and have significantly lower odor thresholds than NH_3_ which also contributes to odor in the farm environment [20, 21]. Therefore, reducing NH_3_ and H_2_S which represent odor gas generated from the layer farm besides mitigating the environmental problems will also have a positive effect on the intestinal health of the animal. Nutritional manipulations, for example, the inclusion of probiotics or organic acids in the diet, have been used to regulate cecal microbiota for mitigating odor emission in animals [22]. Several recent studies have explored the associations between intestinal microbiota and performance traits in chickens and found that the gut microbial structure influences feed conversion efficiency and body weight gain [2, 23–28]. However, limit studies have been conducted to explore the relationship between gut microbiota and odor emission in laying hens when taking breed into consideration. A deeper understanding of the impact and mechanisms of cecal microbiota influencing odor production in laying hens could contribute to the breeding and selection of environmentally friendly laying hens and the development of more effective strategies to optimize an intestinal microbiota structure that would achieve more efficient odor mitigation and productivity improvements.

Based on the available information, we postulated that the gut microbiota structure, which differs among breeds, influences enteric odor production in chickens. The present study quantified and characterized the odor production of two most widely reared laying hen breeds, namely, Hy-Line Gray and Lohmann Pink, and investigated the population structure of their microbiota using 16S rRNA sequencing and q-PCR technology.

## Results

### Production performance and odor production

**Fig. 1:**
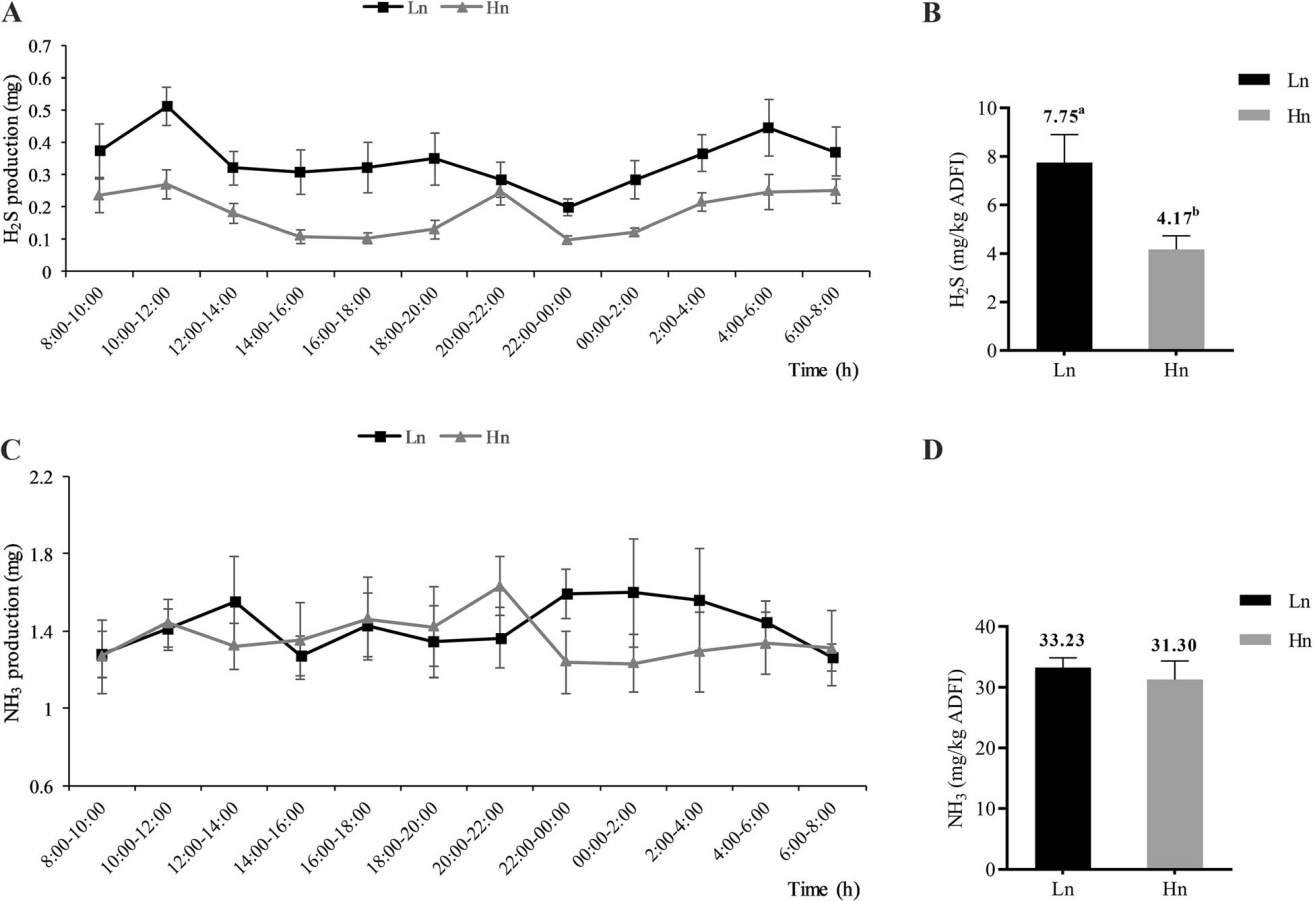
The production of H_2_S and NH_3_ at different times and on the whole day. **a** The production of H_2_S every 2 h, each value is a mean for 3 days (6 replicates). **b** Cumulative daily H_2_S production per kg average daily feed intake of chickens (ADFI). **c** The production of NH_3_ every 2 h, each value is a mean for 3 days (6 replicates). **d** Cumulative daily NH_3_ production per kg ADFI. Error bars show one standard deviation and the mean value was labeled above the error bars with different superscripts mean significant difference (*P* < 0.05). Hn: Hy-Line, Ln: Lohmann

The body weight, ADFI, laying rate, average egg weight and feed-egg ratio of the hens were not significantly different between the two breeds, which eliminated the confounding effects on odor gas production these performance traits divergences might exert (*P* > 0.05) (Additional file 1: Table S3). The recovery rates of the 12 chambers were between 83.85 to 96.62% and 83.01 to 95.31% for NH_3_ and H_2_S respectively (Additional file 1: Table S4). Due to the good recovery rate, these 12 chambers could be used for odor measurement. The total daily H_2_S production per kg average daily feed intake of Hy-Line Gray was significantly lower than that of Lohmann Pink (7.75 vs. 4.17 mg/kg ADFI for Lohmann and Hy-Line, respectively) (*P <* 0.05) (Fig. 1a, b). However, no significant difference was observed in total daily NH_3_ production per kg average daily feed intake between two breeds (33.23 vs. 31.30 mg/kg ADFI) (*P* > 0.05) (Fig. 1c, d).

### Profile characteristics in cecal content

**Fig. 2:**
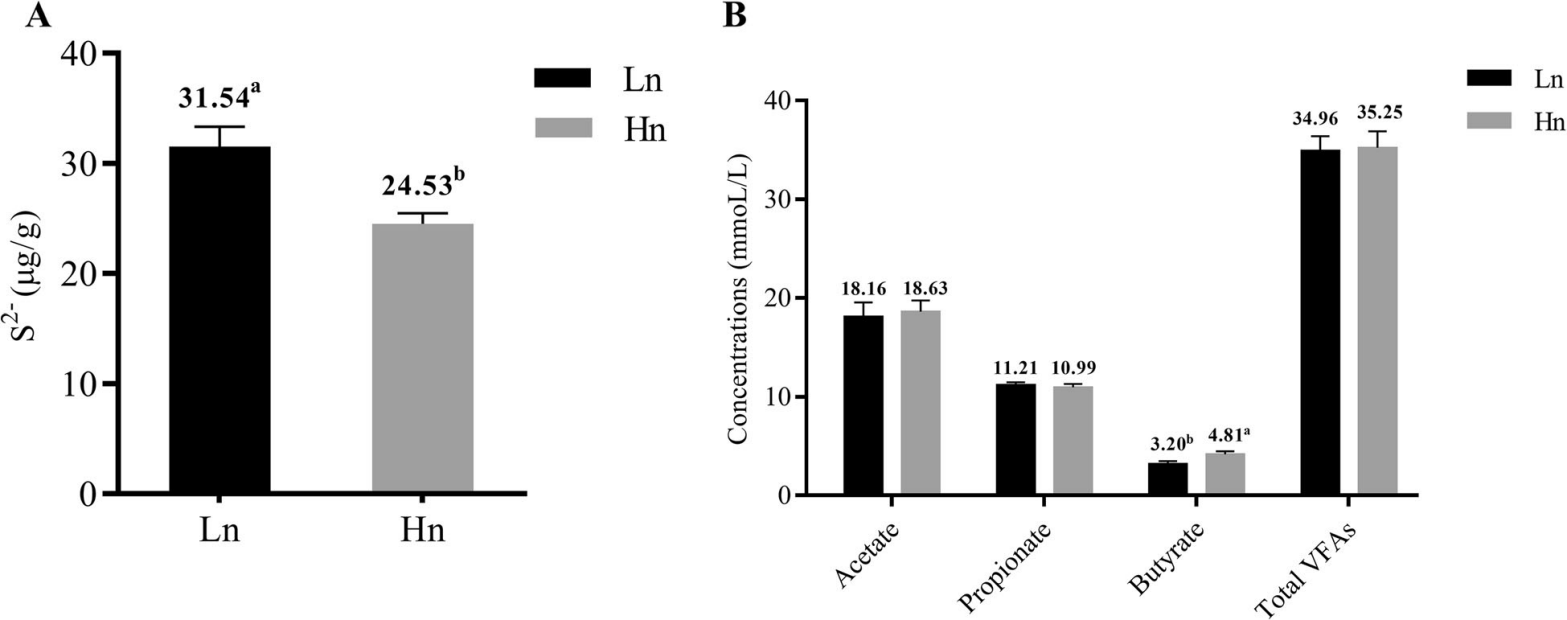
The concentrations of soluble sulfide (S^2−^) and VFAs in the cecal content. **a** The concentration of soluble sulfide (S^2−^). **b** The concentration of VFAs. Error bars show one standard deviation, and the mean value was calculated from 30 replicates. The mean value is labeled above the error bars with different superscripts indicating a significant difference (*P* < 0.05). Hn: Hy-Line, Ln: Lohmann

The pH value and concentrations of ammonium, soluble sulfide (S^2−^), sulfate radical (SO_4_^2−^) and VFAs in cecal content were determined. Compared to Lohmann, Hy-Line showed markedly lower concentrations of soluble sulfide (31.54 vs. 24.53 μg/g) but higher concentrations of butyrate (3.20 vs. 4.81mmoL/L) in the cecal content (Fig. 2a, b) (*P <* 0.05). However, the pH value and the concentrations of ammonium, sulfate radical, acetate, propionate and Total VFAs in cecal content were not different (*P* > 0.05) (Additional file 1: Table S5).

### Microbiota sequencing

After quality processing, a total of 4,641,615 clean sequences were obtained from 60 cecal content samples with an average of 77,360 reads per sample. The sequences were further clustered into an average of 674 operational taxonomic units (OTUs). Good’s coverage was greater than 99.7%, indicating that most of the OTUs present in the samples were detected and the sampling depth was sufficient to characterize the microbiota in cecal content samples (Additional file 1: Figure S2).

### Microbial diversity and relative abundance

**Fig. 3:**
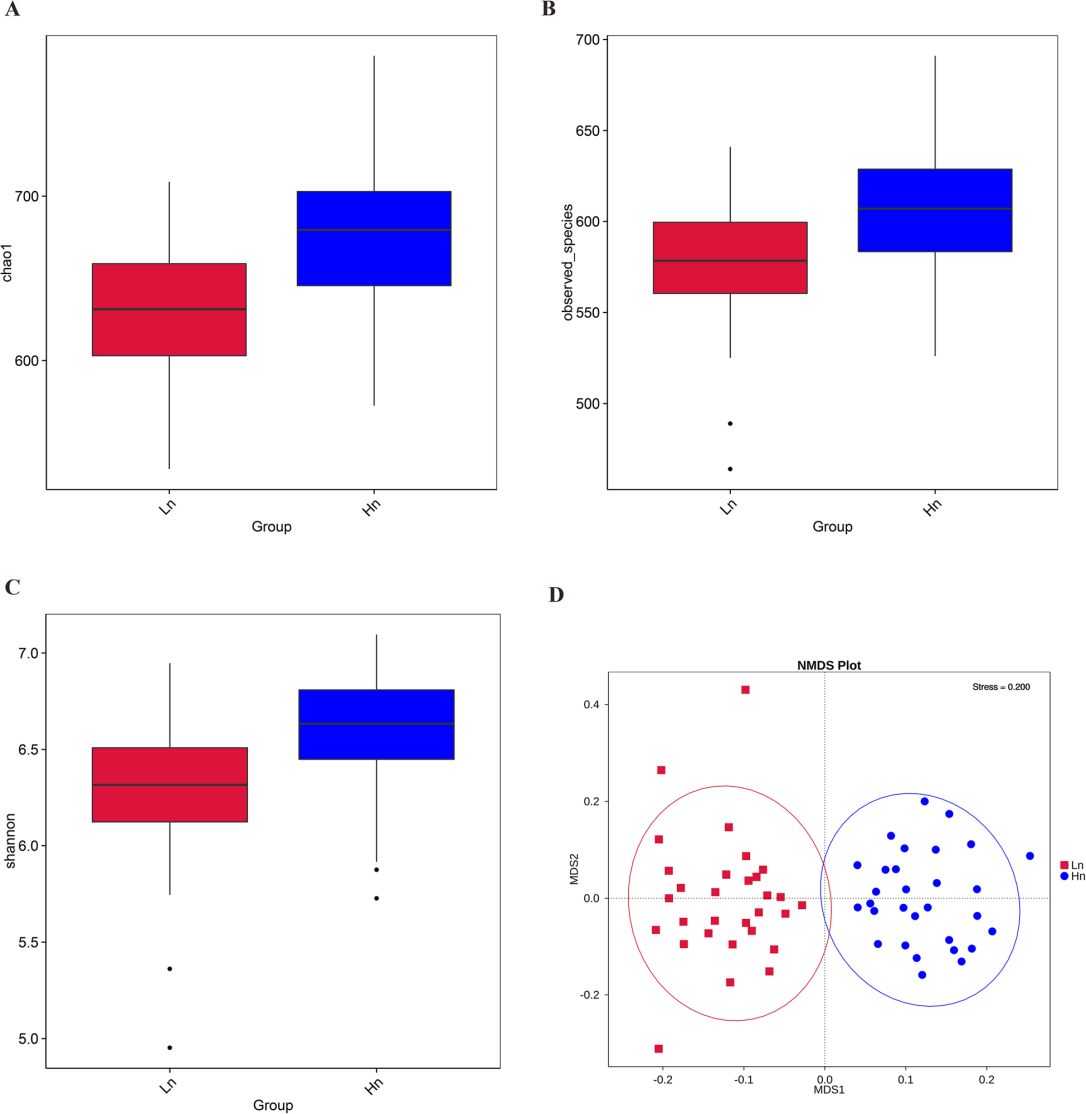
Chao1, Observed species and Shannon index box plots of two groups and NMDS analysis for each individual chicken sample. **a**, **b** Chao1 and Observed species index with a box plot exhibiting the species abundance difference. **c** The Shannon index with a box plot exhibiting the community diversity. **d** NMDS was performed on OTU matrices; each point represents a sample, and two cluster indicated a significant difference of community structure between two samples

**Fig. 4:**
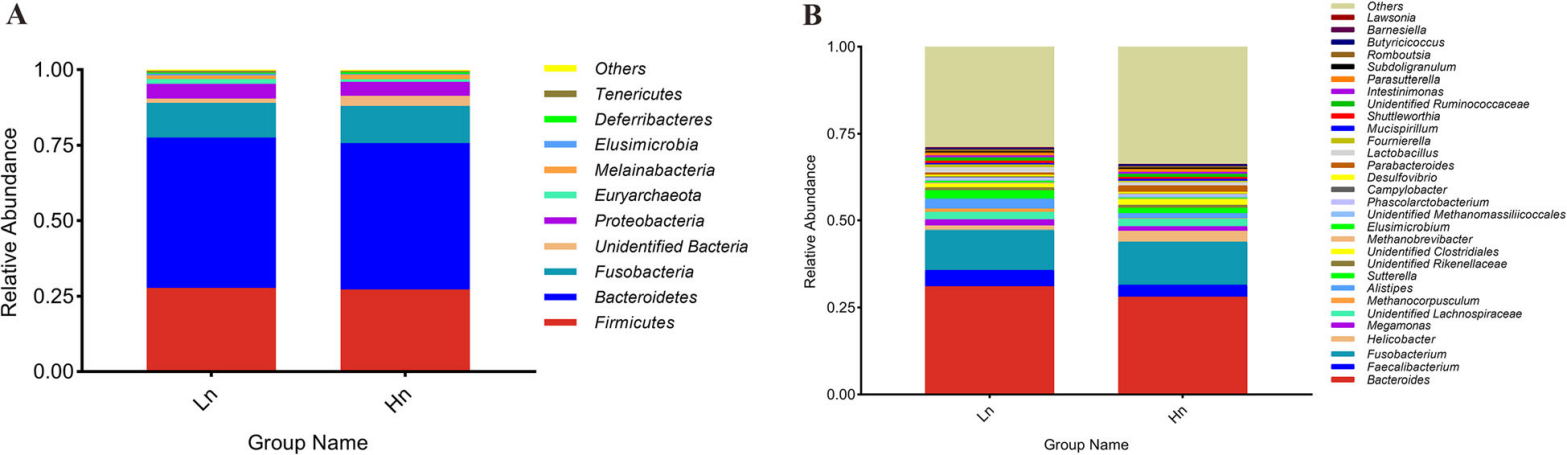
The relative abundance of bacteria in chicken ceca at the phylum and genus levels. **a** Only the top 10 taxa were plotted at the phylum level. **b** Only the top 30 taxa were plotted at genus level. Hn: Hy-Line, Ln: Lohmann

**Fig. 5:**
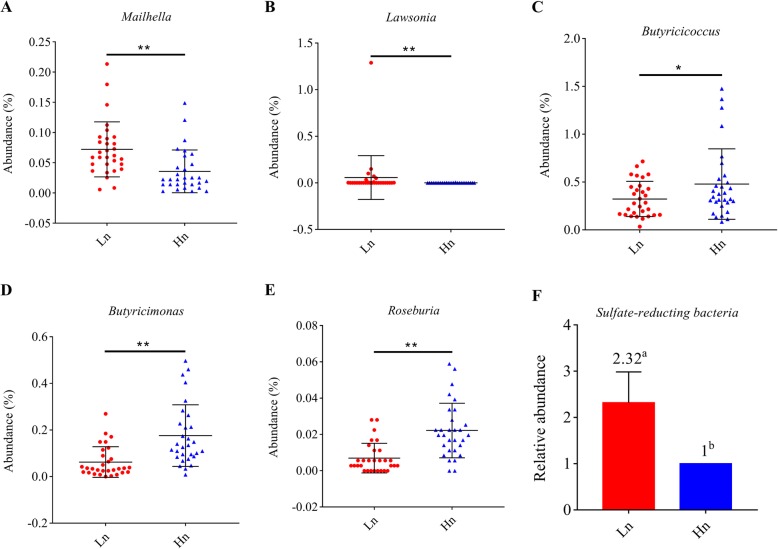
The relative abundance of significantly different genera related to H_2_S and butyrate production. **a**, **b** The abundance of H_2_S production-related bacteria. **c-e** The abundance of butyrate production-related bacteria. **f** The abundance of SRB was determined by q-PCR and considering the abundance of SRB in Hn as 1. Each point represents a sample; error bars show one standard deviation, calculated from 30 replicates. * and a superscript letter indicate significant difference. Hn: Hy-Line, Ln: Lohmann

The Chao1 and Observed species indices are indicators for species abundance, while the Shannon index is applied to evaluate the diversity of gut microbiota. Comparative analysis between breeds revealed that Hy-Line had a higher taxa abundance and diversity than Lohmann, as indicated by significantly different Chao1, Observed species and Shannon indices (*P <* 0.01) (Fig. 3a-c). Between-group diversity was assessed using nonmetric multidimensional scaling (NMDS) analysis, the bacterial community profiles clustered into two groups, highlighting significant differences between two breeds (Fig. 3d). *Bacteroidetes* (mean relative abundance: 49.9 and 48.4% for Lohmann and Hy-Line, respectively), *Firmicutes* (27.8 and 27.3%) and *Fusobacteria* (11.5 and 12.4%) were the most abundant bacteria phyla in the cecum of laying hens. Besides, *Proteobacteria* (4.9 and 4.7%), *Euryarchaeota* (1.6 and 0.9%), *Melainabacteria* (1.1 and 1.4%) and Unidentified *Bacteria* (1.4 and 3.3%) also had a high relative abundance (> 1%) (Fig. 4a). The proportion of Unidentified *Bacteria* but not the other six dominant phyla were significantly different between two breeds (Hy-Line> Lohmann, *P* = 0.007). At the genus level, nine genera were determined with > 1% relative abundance in both two breeds. *Bacteroides* (mean relative abundance: 31.0 and 28.0% for Lohmann and Hy-Line, respectively), *Fusobacterium* (11.5 and 12.4%) and *Faecalibacterium* (4.7 and 3.5%) were found to be most common in the two breeds (Fig. 4b). Furthermore, *Helicobacter* (1.2 and 3.1%), *Megamonas* (1.8 and 1.2%), Unidentified *Lachnospiraceae* (2.2 and 2.1%), *Alistipes* (2.8 and 1.5%), *Sutterella* (2.4 and 1.6%) and Unidentified *Clostridiales* (1.1 and 1.6%) also accounted for the predominant genera. Among them, the abundance of *Helicobacter* (Hy-Line>Lohmann, *P* = 0.003), *Sutterella* (Lohmann>Hy-Line, *P* = 0.008) and *Alistipes* (Lohmann>Hy-Line, *P* = 0.002) were significantly different between two breeds. In total, 42 genera showed significant differences between the two breeds but the abundance of most different genera is less than 1% (Additional file 1: Table S6). We focused on some bacteria which related to H_2_S and butyrate production and found that *Mailhella* (0.07 and 0.04%, *P* = 0.001) and *Lawsonia* (0.06 and 0.0004%, *P* = 0.003) were significantly more abundant in Lohmann, whereas the relative abundance of *Butyricicoccus* (0.32 and 0.48%, *P* = 0.035), *Butyricimonas* (0.06 and 0.18%, *P* = 0.001), and *Roseburia* (0.007 and 0.02%, *P* = 0.001) were significantly higher in Hy-Line (Fig. 5a-e). In next step, we conducted correlation analysis between odor production and the abundance of odor-related bacteria (Additional file 1: Table S7), and found that the abundance of *Desulfovibrio*, *Mailhella*, and *Lawsonia* had a significantly positive correlation with H_2_S production, whereas the abundance of *Butyricicoccus*, *Butyricimonas* and *Roseburia* had a significantly negative correlation with H_2_S production (*P <* 0.05). In addition, the abundance of *Fusobacterium* and Unidentified *Clostridiales* had a significantly positive correlation with NH_3_ production (*P <* 0.05).

### The relative abundance of the *aprA* gene between breeds

In consideration of the limitation of 16S rRNA sequencing on quantifying the abundance of SRB. We conducted Real-time PCR to detect the relative abundance of specific functional *aprA* gene of SRB in cecal content. Compared to Hy-Line, Lohmann had a higher relative abundance of SRB functional *aprA* gene (2.32 vs. 1) (*P* < 0.05), indicating that the relative abundance of SRB in Lohmann are significantly higher than Hy-Line (Fig. 5f).

### Predictive functions of the microbial community

**Fig. 6:**
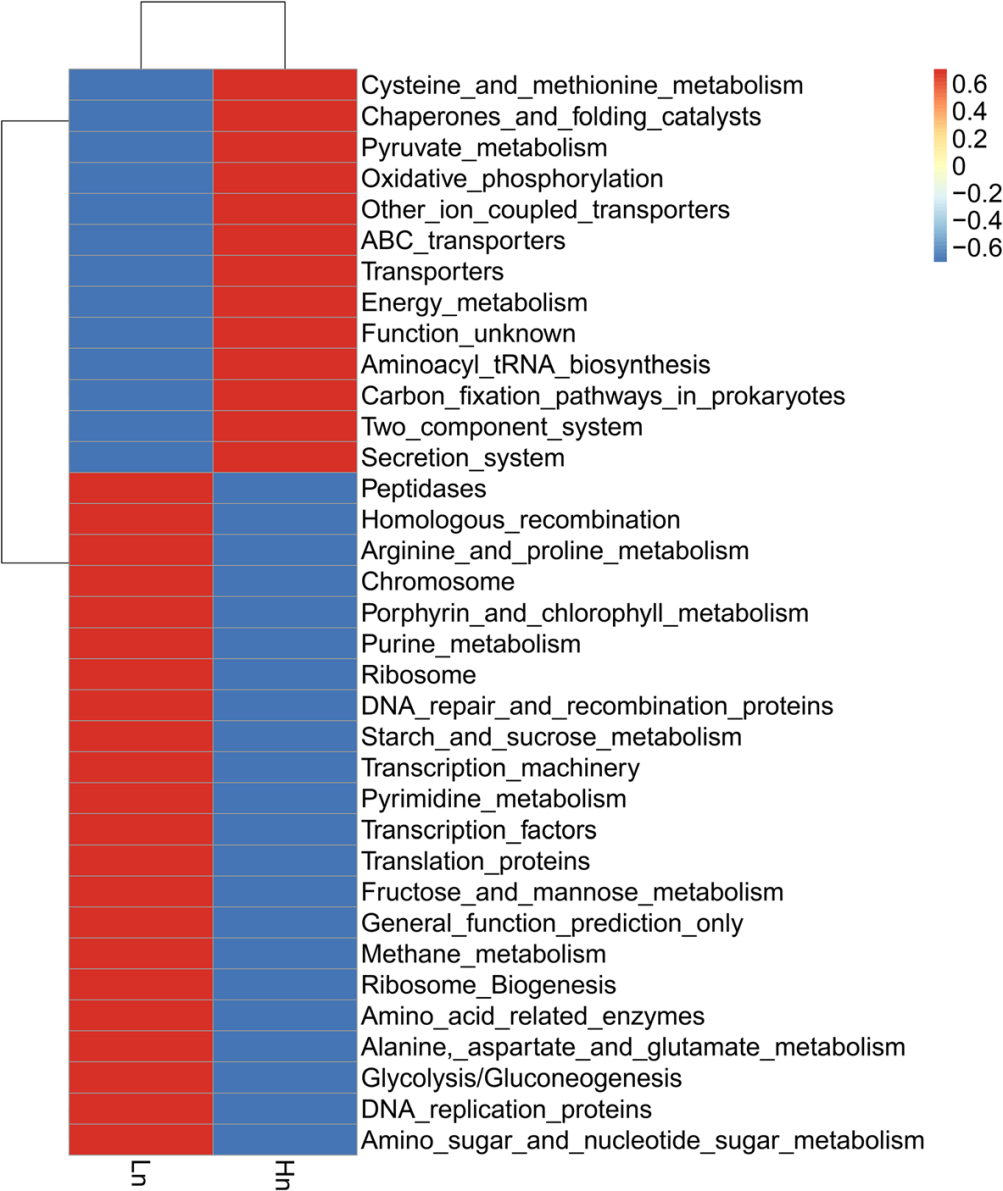
Significantly different pathways between two breeds based on PICRUSt functional prediction. A red color refers to a higher enrichment whereas a blue color refers to a lower enrichment in the bacterial metabolism pathway. Hn: Hy-Line, Ln: Lohmann

Based on the PICRUSt functional prediction, 35 pathways were shown to achieve significant differences at KEGG level 3 (Fig. 6). Thirteen pathways were upregulated in the microbial community of Hy-Line whereas 22 pathways were upregulated in the microbial community of Lohmann. The majority of these pathways are involved in substance metabolism. We focused on some pathways which related to carbohydrate metabolism and protein metabolism. Notably, a significant elevation in oxidative phosphorylation, pyruvate metabolism, energy metabolism, two component system, secretion system, but a decline in amino acid related enzymes, arginine and proline metabolism, and alanine-aspartate and glutamate metabolism were observed in Hy-Line.

### Odor production and profile characteristics in fermentation broth

**Fig. 7:**
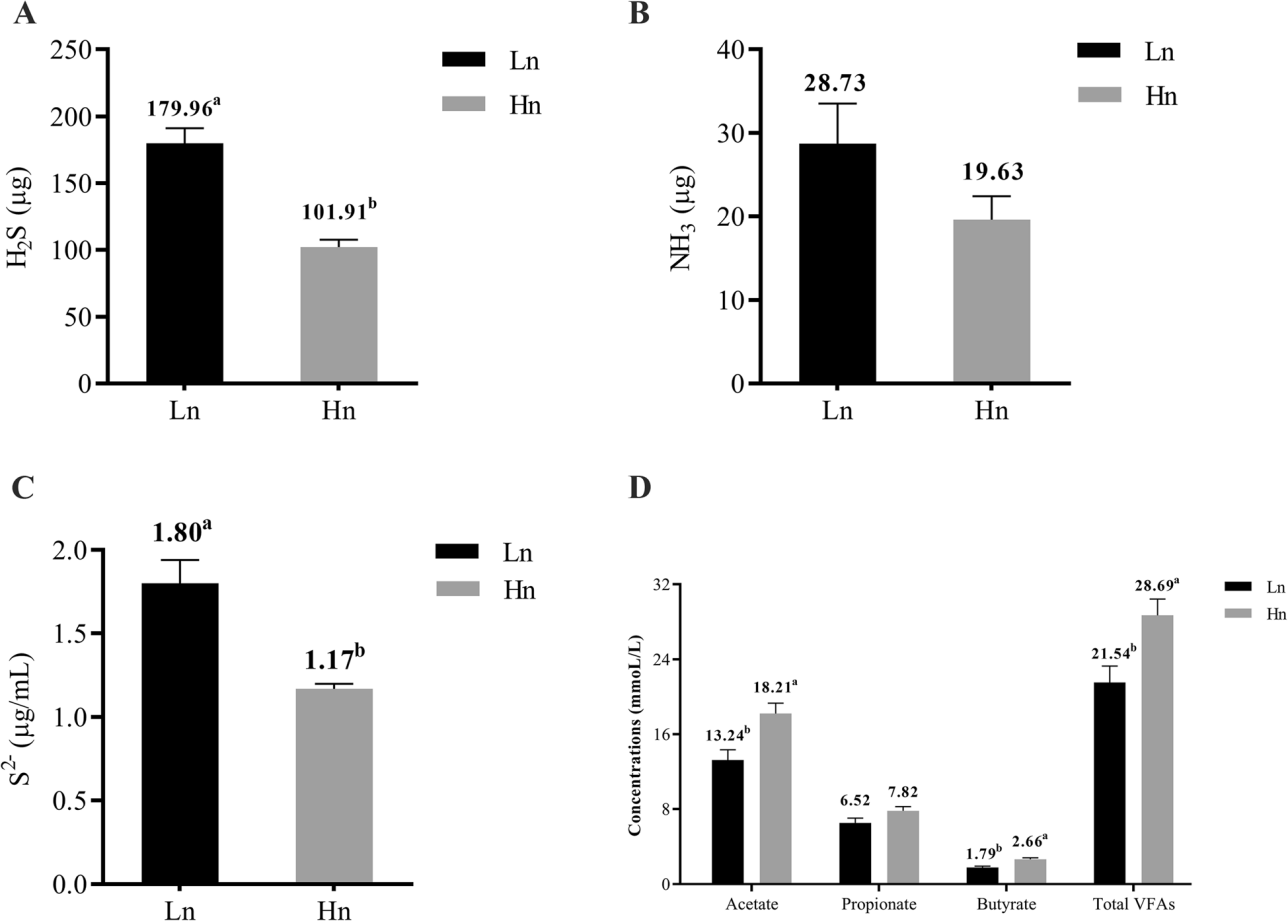
Odor production and profile characteristics in fermentation broth. **a** The production of H_2_S from fermentation. **b** The production of NH_3_ from fermentation. **c** The concentration of soluble sulfide (S^2−^) in fermentation broth. **d** The concentration of VFAs in fermentation broth. Error bars show one standard deviation, and the mean value was calculated from six replicates. The mean value is labeled above the error bars with different superscripts indicating a significant difference (*P* < 0.05). Hn: Hy-Line, Ln: Lohmann

We found that different H_2_S production between the two breeds may result from dissimilar cecal microbiota structure. Then we extracted the microbial flora from the cecum of laying hens and conducted in vitro fermentation trial to further verify the relationship between microbiota structure and H_2_S production. Similarly, lower H_2_S production (179.96 vs. 101.91 μg) was observed in the Hy-Line (*P <* 0.05) (Fig. 7a), but NH_3_ production (28.73 vs. 19.36 μg) did not differ between two groups (*P* > 0.05) (Fig. 7b). Lower concentrations of soluble sulfide (1.80 vs. 1.17 μg/mL) and but higher concentrations of acetate (13.24 vs. 18.21 mmoL/L), butyrate (1.79 vs. 2.66 mmoL/L) and total VFAs (21.54 vs. 28.69 mmoL/L) were observed in the Hy-Line fermentation broth (*P <* 0.05) (Fig. 7c, d). In addition, lower concentrations of sulfate radical and ammonium were also observed in the Hy-Line fermentation broth (*P <* 0.05), but the pH value and the concentrations of propionate in fermentation broth did not differ between two breeds (*P* > 0.05) (Additional file 1: Table S5).

## Discussion

Within host species, factors such as diet and feeding regime could affect the population structure of chicken microbiota [29]. Investigating the role of breed in microbiota structure requires adequate control of confounding effects that may be caused by other factors. Thus, we selected only the two most commonly used laying hen breeds (Hy-Line and Lohmann) and strictly maintained them in the same location and with the same feeding regime. The comparison of microbiota composition between chicken breeds can provide valuable insight into the association between microbiota composition and odor emission.

Based on the principle of the respiration chambers for pigs that we previously constructed and used to measure enteric methane production [30–32], we constructed 12 respiration chambers for laying hens. In the present study, a lower H_2_S production and lower concentration of soluble sulfide in the cecal content were shown in Hy-Line hens compared to those of Lohmann hens (Figs. 1 and 2a), these findings were in agreement with a previous report that the concentrations of gut soluble sulfide and sulfate radical are positively correlated with the release of hydrogen sulfide [33]. In addition, a higher production of butyrate in the Hy-Line cecal content indicated that active carbohydrate metabolism may exist in the Hy-Line cecum (Fig. 2b). However, odor production and cecal profiles can also be influenced by the nature of the substrates flowing out from the small intestine. Fermentation compounds from the distal ileum (into cecum) were probably different as a consequence of their different foregut digestibility between the two breeds. To better explain the role of cecal microbiota in the different odor production and cecal profiles, we conducted a follow-up in vitro fermentation experiment to exclude the possible confounding effects that may be caused by any differences in foregut digestibility between the two breeds. The results of the latter experiment are also in accordance with the higher production of H_2_S and soluble sulfide in Lohmann from the in vivo trial (Fig. 7a, b). Taken together, these results indicate that Hy-Line has a microbial structure that favors lower H_2_S production and soluble sulfide concentrations but higher VFA production. Interestingly, we did not find a significant difference in NH_3_ production and ammonium concentration (except for a lower concentration of ammonium in the Hy-Line incubation broth) in our in vivo and in vitro experiments. We speculate that there may be no notable differences in the abundance of some dominant NH_3_ producing bacteria between the two breeds.

To validate the hypothesis that differences in odor production and cecal profiles are associated with different microbiota structures and microbial metabolic activities between the two breeds, we further compiled the 16S rRNA gene amplicon sequencing data and used the computation tool PICRUSTs to predict microbial community functions. In agreement with previous studies [34–36], *Bactemidetes*, *Firmicutes* and *Fusobacteria* were the most predominant phyla in both breeds. In this study, we focused on the major differences in microbiota involved in H_2_S genesis and butyrate production. Most SRB belong to the family *Desulfovibrionaceae* which can use preliminary degraded organic substances as electron donors to reduce sulfate to produce H_2_S [37]. Our 16S data only annotated four genera (*Desulfovibrio*, *Mailhella*, *Bilophila* and *Lawsonia*) belonging to family *Desulfovibrionaceae*. These H_2_S-related bacteria were found to be positively correlated with H_2_S production (Additional file 1: Table S7), and it showed a significantly higher abundance of *Mailhella* and *Lawsonia* in the cecum of Lohmann, although these two genera are quantitatively small in cecum (Fig. 5a, b). In consideration of the inadequate annotation information form 16S rRNA sequencing data due to the low abundance of SRB in the gut (approximately 0.028–0.097%) [17]. We further quantified the functional gene *aprA* that encodes key enzymes of the dissimilatory sulfate-reduction, which is appropriate to determine the number of SRB in the gut [38]. The relative quantification of SRB, to some extent, was consistent with the results of 16S rRNA sequencing (Fig. 5f). It can be inferred that a lower presence of SRB in the Hy-Line hens resulted in lower H_2_S production. Some of the identified differential genera, such as *Butyricicoccus*, *Butyricimonas* and *Roseburia*, are known butyrate producers [39]. We found that these butyrate producers were negatively correlated with H_2_S production and were more abundant in the Hy-Line (Fig. 5c-e), which is in line with the fact that the dissimilatory sulfate-reduction of SRB will compete with butyrate-producing bacteria and that competitive inhibition of SRB exerted by these acid-producers can indirectly mitigate the production of H_2_S [40]. Some genera, such as *Fusobacterium* (11.4 and 12.4%, *P* = 0.712), Unidentified *Clostridiales* (1.1 and 1.6%, *P* = 0.052) and *Campylobacter* (0.2 and 0.1%, *P* = 0.467), have strong urease activity and are considered to be typical NH_3_ producers [41, 42]. We demonstrated strong links between the abundance of these bacteria and NH_3_ production. However, no significant differences in the abundance of these bacteria explained the similar NH_3_ production between the two breeds. Based on our results, these bacteria may become important regulation targets for NH_3_ emission reduction in future research.

Based on the PICRUSt analysis, some pathways associated with energy extraction and provision (including oxidative phosphorylation, pyruvate metabolism, energy metabolism), bacterial colonization and proliferation (including two component system and secretion system) were overrepresented in Hy-Line (Fig. 6). These pathways are crucial for substrate sensing and foraging, as well as carbohydrate substrate transport and utilization [2], implying that a more active carbohydrate metabolism existed in the Hy-Line cecum. In contrast, the majority of amino acid metabolism-associated pathways (including amino acid related enzymes, arginine and proline metabolism, and alanine-aspartate and glutamate metabolism but not cysteine and methionine metabolism) were suppressed in Hy-Line. Active carbohydrate fermentation could inhibit the fermentation of protein, but suppressed protein metabolism in Hy-Line did not lead to lower NH_3_ production. The reason for this discrepancy may be that the production of NH_3_ mainly depends on the hydrolysis of uric acid instead of the fermentation of protein [16, 43], thus a nonsignificant difference in NH_3_ producing bacteria between the two breeds resulted in similar NH_3_ production. In addition, due to the limitation of 16S rRNA for functional prediction, we did not annotate and attain pathways that were directly related to bacterial sulfur metabolism. The state of microbiota and relationships with their colonized ecosystem could be more accurately examined using deep-sequencing technologies such as the metatranscriptome and metaproteome.

## Conclusions

In this study, we found that Hy-Line hens exhibited lower H_2_S production as a result of a lower abundance of SRB but a higher abundance of butyrate producers in the cecum compared to Lohmann hens. Our study suggested that, at least in part, different microbiota structures in the cecum of laying hens contributes to dissimilar odor production, which offers a reference for targeted odor mitigation by the enteric manipulation of identified odor-related microbiota.

## Methods

### Experiment 1: in vivo trial

#### Animals and feeding

Approximately 100 Hy-Line Gray and 100 Lohmann Pink chicks were hatched and fed together in a commercial hatchery (Peng Chang Co., Ltd., Shenzhen, China). Upon reaching 28 weeks of age, 30 laying hens from each breed with similar weights (1.70 ± 0.02 and 1.71 ± 0.02, respectively for Hy-Line and Lohmann) were selected and moved to twelve 2 × 1 × 1.2 m respiration chambers with five hens per chamber (replicates) which were placed in an environmentally controlled room for a 21-day experiment. The hens were all fed the same commercial laying hen diet ad libitum (Additional file 1: Table S1). Clean drinking water was also made available at all time to the hens. The hens were kept in a room maintained at 24 °C with a 12 h lighting and 12 h dark management schedule. The 21-day experiment consisted of 18 days of feeding and 3 days of gas collection. Body weight, average daily feed intake (ADFI), laying rate, average egg weight and feed-egg ratio of the hens were recorded and calculated during the 21-day experimental period. It is worth noting that the diet, age, weight, and feeding environment are consistent between two breeds after hatching and during the experimental period in order to minimize the confounding effects these factors might cause.

#### Design of the respiration chambers and gas collection

Twelve respiration chambers were constructed (patent number: 201610091580.9) (Additional file 1: Figure S1). Each chamber was 2.0 m long× 1.0 m wide× 1.2 m high and constructed using a metal frame covered with transparent poly methyl methacrylate sheets except for the bottom which was made of a steel sheet. Each chamber was fitted with two doors in the front: one for feeding, egg collection and placing and removing the hens and the other for feces removal. Drinking water was provided using a drinker nipple located at the front of the chamber. The respiration chamber was ventilated using an electrical pump (Type: ACO-009D, Hailea, Guangzhou, China) at the outlet end. The ventilation rate was maintained at 40 L/min using a gas flow-meter (Type: LZB-2WB, Specifications: 6–60 L/min, ZhenXing Flowtech, Yuyao, China). The end of the gas outlet was connected to three sampling tubes. The first tube was connected to a bottle of diluted sulfuric acid solution (50 mL of 0.05 mol/L per bottle) for absorption of NH_3_ in the outlet air which flows at 1 L/min flow (Type: LZB-4WB, Specifications: 0.6–6 L/min, ZhenXing Flowtech, Yuyao, China) (Additional file 1: Figure S1, 16). The second tube was connected to a bottle of cadmium sulfate solution (4.3 g 3CdSO_4_·8H_2_O and 0.3 g NaOH and 10 g ammonium alcohol polyvinyl phosphate were diluted to 1 L with water, 10 mL per bottle) for an H_2_S absorption (flow rate of 1 L/min) (Additional file 1: Figure S1, 18). The third tube served to exhaust the remaining outlet air (Additional file 1: Figure S1, 17). The daily gas collection lasted for 24 h with a short intermittent break (approximately 2 min) every 2 h to remove and replace the respective gas absorption solutions. The above procedure was repeated over 3 days (as 3 replicates for the gases produced).

#### Recovery rate of the respiration chambers

The recovery rate of the system was determined based on the procedure of Ji et al. [30]. Standard gas was released into the chamber using a precision reducer and flow meter to ensure a uniform release rate. The standard gas for NH_3_ comprised 1.6% NH_3_ (v/v) and 98.4% (v/v) nitrogen (N_2_). The standard gas for H_2_S comprised 0.02% H_2_S (v/v) and 99.98% (v/v) N_2_. The respiration chamber was ventilated, and samples of outlet air were collected at regular intervals (2 h) as described above. The recovery rate of system (R) was calculated as follows:

$$ \mathrm{R}=\left({\mathrm{M}}_{\mathrm{rec}}/{\mathrm{M}}_{\mathrm{rel}}\right)\times 100\%=\left[{\mathrm{M}}_{\mathrm{rec}}/\left({\mathrm{M}}_{\mathrm{gas}\ \mathrm{bottle}}+{\mathrm{M}}_{\mathrm{gas}\ \mathrm{air}}\right)\right]\times 100\% $$

where M_rec_ is the mass of gas recovered by gas collection measurement (μg); M_rel_ is the mass of gas released (μg); M_gas bottle_ is the mass of gas released by gas bottle (μg); and M_gas air_ is the mass of gas in the inlet air (μg).

#### Measurement of odor production

Gas production (μg) from the chickens every 2 h was calculated as:

$$ \mathrm{M}=\left[\ \left({\mathrm{M}}_{\mathrm{s}}/\mathrm{R}\right)\times 40-{\mathrm{C}}_{\mathrm{a}}\times {\mathrm{V}}_{\mathrm{a}}\right]\times 100\% $$

where M is the mass of gas produced by chickens in 2 h (μg); M_s_ is the mass of gas in the absorption solution (μg); C_a_ is the concentration of gas in the inlet air (μg/L), V_a_ is the volume of inlet air. The ventilation rate (40 L/min) multiplied by 120 min yields the volume of inlet air and was corrected to standard temperature and pressure. R is the recovery rate of gas of the system.

The mass of NH_3_ and H_2_S in solution was measured with a spectrophotometer (Ao Yi technology Co. Ltd., Shanghai, China) based on the Chinese National Environmental Protection Strands (determination of ammonia, Nessler’s reagent spectrophotometry; standard method for hygienic examination of hydrogen sulfide in air of residential areas, methylene-blue spectrophotometric method).

#### Cecal sample collection

At the end of the experiment, all birds were euthanized by cervical dislocation, and then the cecum was ligated and removed from the carcass. The content was aseptically collected into an Eppendorf tube and immediately put in liquid nitrogen and stored at − 80 °C for further experiments.

#### Measurement of cecal profiles

Accurately quantified 1.0 g of cecal content was mixed adequately with 9 mL deionized water in a 15 mL centrifuge tube and then centrifuged at 10,000×g for 5 min, the supernatant was used for measurement. The pH value was determined using a pH meter (INESA Scientific Instrument Co., Ltd., Shanghai, China). The ammonium-nitrogen was determined with a spectrophotometer (Ao Yi technology Co., Ltd., Shanghai, China) based on Nessler’s reagent spectrophotometry method. The sulfate radical (SO_4_^2−^) concentrations were determined using a turbidimetric method according to Deng et al. [21]. The soluble sulfide (S^2−^) concentrations were determined using a methylene-blue colorimetric method. The concentrations of acetate, propionate and butyrate were determined using high-performance liquid chromatography (Agilent Technologies, Inc., California, USA) with an Agilent SB-C18 column (150 × 4.6 mm). Samples were eluted with methanol mixed sodium dihydrogen phosphate buffer (28:72, v/v) at 1 mL/min and were detected with a U.V. monitor at a wavelength of 213 nm.

#### Bacterial DNA extraction, sequence processing and data analysis

Approximately 200 mg of cecal content was used for total DNA extraction using a QIAamp PowerFecal DNA Kit (QIAGEN, Germany) following the manufacturer’s instructions. DNA quantity and quality were measured on a Qubit 2.0 fluorometer (Thermo Fisher scientific, MA, US) and gel electrophoresis. The V3-V4 hypervariable regions of 16S rRNA genes were amplified using primers (forward: 5′-CCTACGGGNGGCWGCAG-3′ and reverse: 5′-GACTACHVGGGTATCTAATCC-3′) [44]. PCR amplifications were carried out using 25 μL reaction mixtures containing 12.5 μL 2X KAPA HiFi HotStart ReadyMix (Kapa Biosystems, UK), 5 μL forward and reverse primers and 2.5 μL DNA template. Amplicons were performed using the following conditions: denaturation at 94 °C for 5 min, 30 cycles of amplification including denaturation (94 °C, 30 s), annealing (50 °C, 30 s), and elongation (72 °C, 30 s), and final elongation at 72 °C for 10 min. The final products were sequenced using an Illumina MiSeq platform at Novogene Co. Ltd. (Tianjin, China).

Raw reads were uploaded into QIIME software v2.1 and quality trimmed. Reads containing ambiguous bases, a low quality score (a quality score lower than 25), or reads shorter than 200 bp were discarded. Trimmed sequences were clustered into 97% similarity OTUs. Annotation assignment and richness analysis of reference OTU sequences were performed by mapping against the Greengenes database with a 0.5 confidence threshold and were taxonomically identified to the genus level. Analysis of alpha diversity (Observed species, Chao 1 index and Shannon index) as well as Good’s Coverage estimates were calculated in QIIME. For beta diversity analysis, NMDS was used to assess the differences in microbiota structure. Comparative analysis for taxa was performed where the Benjamini-Hochberg false discovery rate (FDR) was used for multiple test corrections to minimize FDR during group comparative analyses, and *P*-values less than 0.05 were considered statistically significant. Predictive functions were characterized using Phylogenetic Investigation of Communities by Reconstruction of Unobserved States (PICRUSt) based on the Kyoto Encyclopedia of Genes and Genomes database (KEGG) [45].

#### Q-PCR of the functional gene (adenosine-5′-phosphosulfate reductase alpha subunit gene, *aprA*) of SRB

We further determined the relative abundance of SRB between the two breed groups through their function marker gene *aprA* using q-PCR with the 16S rRNA gene as the reference gene [46, 47] (Additional file 1: Table S2). The total DNA of the bacteria was extracted as described above. The reaction mixtures contained 10 μL SYBR® Green PCR Master Mix (QIAGEN, Germany), 0.5 μL of each primer, 1 μL of DNA samples and 8 μL H_2_O. The q-PCR cycle was set as follows: initial denaturation for 2 min at 95 °C, followed by 35 cycles of denaturing for 15 s at 95 °C, annealing for 30 s at 60 °C, and elongating for 25 s at 72 °C. The relative abundance of the *aprA* gene was compared using the 2^-∆∆CT^ value.

### Experiment 2: in vitro fermentation trial

To better explain the impact of cecal microbiota on the differences in H_2_S production, a follow-up in vitro fermentation study was conducted with 2 inocula × 6 replicates (12 syringes) plus two blanks, making a total of 14 syringes.

#### Preparation of the inoculum and fermentative substrate

Twenty Hy-Line Gray and twenty Lohmann Pink laying hens at 28 weeks of age were sacrificed, and the cecum was ligated immediately. The cecal contents in the same breed group were pooled, thoroughly mixed and diluted in sodium and ammonia bicarbonate buffer solution (35.0 g NaHCO_3_ + 4.0 g NH_4_HCO_3_ for a 1 L volume) at a ratio of 1:3 (W/V) according to the method of Wang et al. [16]. The intestinal content–buffer mixture was stirred for 60 s in a blender after which the solution was squeezed through four layers of surgical gauze, and then mixed with the buffer mineral solution at a 1:2 ratio (V/V) at 40 °C under continuous flushing with CO_2_. A corn-soybean basal layer diet was used as a substrate for the fermentation.

#### In vitro fermentation

Approximately 30 mL of the inoculum was added to each 100-mL gas syringe with 0.5 g of the substrate. After removing the air from the head-space of the syringe, the syringe was sealed with a clip and placed in a 40 °C incubator rotated at 60 rpm for 12 h. The volume of gas produced at the head-space was recorded. At the end of incubation, the syringes were put into an ice box to stop further fermentation, and the gas was transferred by syringes into gas collection bag for NH_3_ and H_2_S analysis. Ten milliliter fermentation broth was sampled and stored at − 80 °C for chemical analysis. The quantity of NH_3_ in the syringes and the concentrations of ammonium-nitrogen in fermentation broth were determined using Nessler’s reagent spectrophotometry method. The quantity of H_2_S in the syringes and the concentrations of soluble sulfide (S^2−^) in the fermentation broth were determined using the methylene-blue colorimetric method. The pH value was determined using a pH meter (INESA Scientific Instrument Co., Ltd., Shanghai, China). The concentration of sulfate radical (SO_4_^2−^) was determined using the turbidimetric method. The concentrations of VFAs were determined using high-performance liquid chromatography as described in Experiment 1(“Measurement of cecal profiles section”).

### Statistical analysis

The significance of the data was analyzed with an independent-samples t-test using the commercially available statistical software package SPSS 22.0 (SPSS, Chicago, IL, USA). Differences were considered significant if *P* < 0.05. Data are presented as the means with their standard errors. The correlation analysis was conducted with Pearson correlation-based model using SPSS 22.0.

## Supplementary information

**Additional file 1:** **Table S1.** Diet composition and nutrient levels. **Table S2.** The primer sequences of the *aprA* gene and 16S rRNA gene. **Table S3.** The production performance of laying hens. **Table S4.** The recovery of respiration chamber system. **Table S5.** Profile characteristics in cecal content and fermentation broth. **Table S6.** The relative abundance of the 42 significantly different genera between two groups. **Table S7.** Correlation coefficients of some odor-related microbiota and odor production. **Figure S1.** The structure of respiration chamber system. **a** The physical picture of respiration chamber. **b** Absorption bottle for gas collection. c The sketch of respiration chamber. 1 The inner cage, 2 Feed trough, 3 Inner floor, 4 Eggs trough, 5 Drinker, 6 fan, 7 PVC floor, 8 Organic glass cover, 9 Baffle of feeding, 10 Feces collection, 11 Baffle of feces collection, 12 Air inlet, 13 Air outlet, 14 Total air outlet, 15 Air pump, 16 Ammonia absorption bottle, 17 Exhaust hose, 18 Hydrogen sulfide absorption bottle, 19 Flowmeter. **Figure S2.** The Good’s coverage index box plots of two groups. Good’s coverage index with a box plot exhibiting the depth of 16S rRNA sequencing. Hn: Hy-Line, Ln: Lohmann.

## Data Availability

The 16S rRNA datasets generated and analysed during the current study are available in the European Nucleotide Archive database under the accession number PRJEB30541.

## Abbreviations

ADFI: Average daily feed intake
GIT: Gastrointestinal tract
H_2_S: Hydrogen sulfide
NH_3_: Ammonia
S^2−^: Soluble sulfide
SO_4_^2−^: Sulfate radical
SRB: Sulfate-reducing bacteria
VFAs: Volatile fatty acids

## References

[1] McWhorter TJ, Caviedes-Vidal E, Karasov WH (2009). The integration of digestion and osmoregulation in the avian gut. Biol Rev.

[2] Hou Q, Kwok LY, Zheng Y, Wang L, Guo Z, Zhang J (2016). Differential fecal microbiota are retained in broiler chicken lines divergently selected for fatness traits. Sci Rep.

[3] Gong J, Si W, Forster RJ, Huang R, Yu H, Yin Y (2007). 16S rRNA gene-based analysis of mucosa-associated bacterial community and phylogeny in the chicken gastrointestinal tracts: from crops to ceca. FEMS Microbiol Ecol.

[4] Videnska P, Faldynova M, Juricova H, Babak V, Sisak F, Havlickova H (2013). Chicken faecal microbiota and disturbances induced by single or repeated therapy with tetracycline and streptomycin. BMC Vet Res.

[5] Stanley D, Hughes RJ, Moore RJ (2014). Microbiota of the chicken gastrointestinal tract: influence on health, productivity and disease. Appl Microbiol Biotechnol.

[6] Oakley BB, Lillehoj HS, Kogut MH, Kim WK, Maurer JJ, Pedroso A (2014). The chicken gastrointestinal microbiome. FEMS Microbiol Lett.

[7] Sergeant MJ, Constantinidou C, Cogan TA, Bedford MR, Penn CW, Pallen MJ (2014). Extensive microbial and functional diversity within the chicken cecal microbiome. PLoS One.

[8] Brisbin JT, Gong J, Sharif S (2008). Interactions between commensal bacteria and the gut-associated immune system of the chicken. Anim Health Res Rev.

[9] Lei F, Yin Y, Wang Y, Deng B, Yu HD, Li L (2012). Higher-evel production of volatile fatty acids in vitro by chicken gut microbiotas than by human gut microbiotas as determined by functional analyses. Appl Environ Microbiol.

[10] Qu A, Brulc JM, Wilson MK, Law BF, Theoret JR, Joens LA (2008). Comparative metagenomics reveals host specific metavirulomes and horizontal gene transfer elements in the chicken cecum microbiome. PLoS One.

[11] Danzeisen JL, Kim HB, Isaacson RE, Tu ZJ, Johnson TJ (2011). Modulations of the chicken cecal microbiome and metagenome in response to anticoccidial and growth promoter treatment. PLoS One.

[12] McCubbin DR, Apelberg BJ, Roe S, Divita F (2002). Livestock ammonia management and particulate-related health benefits. Environ Sci Technol.

[13] Xin H, Gates RS, Green AR, Mitloehner FM, Moore PA, Wathes CM (2011). Environmental impacts and sustainability of egg production systems. Poult Sci.

[14] Zhang Y, Dore AJ, Ma L, Liu XJ, Ma WQ, Cape JN (2010). Agricultural ammonia emissions inventory and spatial distribution in the North China plain. Environ Pollut.

[15] Dunkley KD, Dunkley CS, Njongmeta NL, Callaway TR, Hume ME, Klubena LF (2007). Comparison of in vitro fermentation and molecular microbial profiles of high-fiber feed substrates incubated with chicken cecal inocula. Poult Sci.

[16] Wang A, Wang Y, Di Liao X, Wu Y, Liang JB, Laudadio V (2016). Sodium butyrate mitigates in vitro ammonia generation in cecal content of laying hens. Environ Sci Pollut R.

[17] Deng YF, Liu YY, Zhang YT, Wang Y, Liang JB, Tufarelli V (2017). Efficacy and role of inulin in mitigation of enteric sulfur-containing odor in pigs. J Sci Food Agric.

[18] Stokstad E (2014). Ammonia pollution from farming may exact hefty health costs. Science.

[19] Hunde A, Patterson P, Ricke S, Kim WK (2012). Supplementation of poultry feeds with dietary zinc and other minerals and compounds to mitigate nitrogen emissions—a review. Biol Trace Elem Res.

[20] Gemert LJV (2003). Compilations of odour threshold values in air, water and other media.

[21] Deng YF, Liao XD, Wang Y, Liang JB, Tufarelli V (2015). Prebiotics mitigate in vitro sulfur-containing odour generation in caecal content of pigs. Ital J Anim Sci.

[22] Lee K, Lillehoj HS, Siragusa GR (2010). Direct-fed Microbials and their impact on the intestinal microflora and immune system of chickens. J Poult Sci.

[23] Ding J, Zhao L, Wang L, Zhao W, Zhai Z, Leng L (2016). Divergent selection-induced obesity alters the composition and functional pathways of chicken gut microbiota. Genet Sel Evol.

[24] Schokker D, Veninga G, Vastenhouw SA, Bossers A, de Bree FM, Kaal-Lansbergen LM (2015). Early life microbial colonization of the gut and intestinal development differ between genetically divergent broiler lines. BMC Genomics.

[25] Mignon-Grasteau S, Narcy A, Rideau N, Chantry-Darmon C, Boscher M, Sellier N (2015). Impact of selection for digestive efficiency on microbiota composition in the chicken. PLoS One.

[26] Meng H, Zhang Y, Zhao L, Zhao W, He C, Honaker CF (2014). Body weight selection affects quantitative genetic correlated responses in gut microbiota. PLoS One.

[27] Zhao L, Wang G, Siegel P, He C, Wang H, Zhao W, et al. Quantitative genetic background of the host influences gut microbiomes in chickens. Sci Rep-Uk. 2013;3(1). 10.1038/srep01163 PMC3557447 23362462

[28] Stanley D, Denman SE, Hughes RJ, Geier MS, Crowley TM, Chen H (2012). Intestinal microbiota associated with differential feed conversion efficiency in chickens. Appl Microbiol Biotechnol.

[29] Kers JG, Velkers FC, Fischer E, Hermes G, Stegeman JA, Smidt H (2018). Host and environmental factors affecting the intestinal microbiota in chickens. Front Microbiol.

[30] Ji ZY, Cao Z, Liao XD, Wu YB, Liang JB, Yu B (2011). Methane production of growing and finishing pigs in southern China. Anim Feed Sci Technol.

[31] Gong YL, Liang JB, Jahromi MF, Wu YB, Wright AG, Liao XD (2018). Mode of action of Saccharomyces cerevisiae in enteric methane mitigation in pigs. Animal.

[32] Cao Z, Gong YL, Liao XD, Liang JB, Yu B, Wu YB (2013). Effect of dietary fiber on methane production in Chinese Lantang gilts. Livest Sci.

[33] Muezzinoglu A (2003). A study of volatile organic sulfur emissions causing urban odors. Chemosphere.

[34] Mohd SM, Sieo CC, Chong CW, Gan HM, Ho YW (2015). Deciphering chicken gut microbial dynamics based on high-throughput 16S rRNA metagenomics analyses. Gut Pathog.

[35] Ding J, Dai R, Yang L, He C, Xu K, Liu S (2017). Inheritance and establishment of gut microbiota in chickens. Front Microbiol.

[36] Mancabelli L, Ferrario C, Milani C, Mangifesta M, Turroni F, Duranti S (2016). Insights into the biodiversity of the gut microbiota of broiler chickens. Environ Microbiol.

[37] Rey FE, Gonzalez MD, Cheng J, Wu M, Ahern PP, Gordon JI (2013). Metabolic niche of a prominent sulfate-reducing human gut bacterium. Proc Natl Acad Sci U S A.

[38] Meyer B, Kuever J (2007). Molecular analysis of the distribution and phylogeny of dissimilatory adenosine-5′-phosphosulfate reductase-encoding genes (aprBA) among sulfur-oxidizing prokaryotes. Microbiology.

[39] Eeckhaut V, Van Immerseel F, Croubels S, De Baere S, Haesebrouck F, Ducatelle R (2011). Butyrate production in phylogenetically diverse Firmicutes isolated from the chicken caecum. Microb Biotechnol.

[40] Marquet P, Duncan SH, Chassard C, Bernalier-Donadille A, Flint HJ (2009). Lactate has the potential to promote hydrogen sulphide formation in the human colon. FEMS Microbiol Lett.

[41] Davila AM, Blachier F, Gotteland M, Andriamihaja M, Benetti PH, Sanz Y (2013). Intestinal luminal nitrogen metabolism: role of the gut microbiota and consequences for the host. Pharmacol Res.

[42] Dai ZL, Wu G, Zhu WY (2011). Amino acid metabolism in intestinal bacteria: links between gut ecology and host health. Front Biosci (Landmark Ed).

[43] Panetta DM, Powers WJ, Lorimor JC (2005). Management strategy impacts on ammonia volatilization from swine manure. J Environ Qual.

[44] Pandit RJ, Hinsu AT, Patel NV, Koringa PG, Jakhesara SJ, Thakkar JR (2018). Microbial diversity and community composition of caecal microbiota in commercial and indigenous Indian chickens determined using 16s rDNA amplicon sequencing. Microbiome.

[45] Langille MGI, Zaneveld J, Caporaso JG, McDonald D, Knights D, Reyes JA (2013). Predictive functional profiling of microbial communities using 16S rRNA marker gene sequences. Nat Biotechnol.

[46] Meyer B, Kuever J (2007). Molecular analysis of the diversity of sulfate-reducing and sulfur-oxidizing prokaryotes in the environment, using aprA as functional marker gene. Appl Environ Microbiol.

[47] Denman SE, McSweeney CS (2006). Development of a real-time PCR assay for monitoring anaerobic fungal and cellulolytic bacterial populations within the rumen. FEMS Microbiol Ecol.

