# Supplementary material for: The microbiota structure in the cecum of laying hens contributes to dissimilar H2S production
Source: BMC Genomics. 2019 Oct 23;20:770. doi: 10.1186/s12864-019-6115-1 (PMC6813079; doi:10.1186/s12864-019-6115-1)
Supplement: Supplementary file 1 — Additional file 1: Table S1. Diet composition and nutrient levels. Table S2. The primer sequences of the aprA gene and 16S rRNA gene. Table S3. The production performance of laying hens. Table S4. The recovery of respiration chamber system. Table S5. Profile characteristics in cecal content and fermentation broth. Table S6. The relative abundance of the 42 significantly different genera between two groups. Table S7. Correlation coefficients of some odor-related microbiota and odor production. Figure S1. The structure of respiration chamber system. a The physical picture of respiration chamber. b Absorption bottle for gas collection. c The sketch of respiration chamber. 1 The inner cage, 2 Feed trough, 3 Inner floor, 4 Eggs trough, 5 Drinker, 6 fan, 7 PVC floor, 8 Organic glass cover, 9 Baffle of feeding, 10 Feces collection, 11 Baffle of feces collection, 12 Air inlet, 13 Air outlet, 14 Total air outlet, 15 Air pump, 16 Ammonia absorption bottle, 17 Exhaust hose, 18 Hydrogen sulfide absorption bottle, 19 Flowmeter. Figure S2. The Good’s coverage index box plots of two groups. Good’s coverage index with a box plot exhibiting the depth of 16S rRNA sequencing. Hn: Hy-Line, Ln: Lohmann. [file 12864_2019_6115_MOESM1_ESM.pdf]

Supplementary material

The microbiota structure in the ceca of laying hens contributes to dissimilar H_2S production

Chun-Bo Huang[1], Lei Xiao[1], Si-Cheng Xing[1], Jing-Yuan Chen[1], Yi-Wen Yang[1], Yang Zhou[1], Wei Chen[1], Juan-Boo Liang[3], Jian-Dui Mi[1,2], Yan Wang[1,2], Yin-Bao Wu[1,2], Xin-Di Liao[1,2] #

[1] College of Animal Science, South China Agricultural University, Guangzhou, China.

[2] Ministry of Agriculture Key Laboratory of Tropical Agricultural Environment, South China Agricultural University, Guangzhou, China.

[3] Institute of Tropical Agriculture, University of Putra Malaysia, Serdang, Malaysia

#Corresponding Author:

Dr. Xin Di Liao

Address: College of Animal Science, South China Agricultural University, Guangzhou, China.

Tel.: +86 20 85280279; E-mail: xdliao@scau.edu.cn

Supplemental Tables:

Table S1 Diet composition and nutrient levels

Ingredients	Content (%)	Nutrient levels	Content
Maize meal	62	ME (MJ/kg)	11.13
Soybean meal	26	CP (%)	16.2
Soybean oil	1	Ca (%)	3.8
Limestone	8	TP (%)	0.45
Monocalcium phosphate	1.54	AP (%)	0.32
Salt	0.3	Lys (%)	0.8
DL-Met	0.16	Met (%)	0.38
Premix [1]	1		
Total	100		

[1] The premix provided the following per kg of diets: Mn 60 mg, Cu 8 mg, Zn 80 mg, Fe 60 mg, I 0.35 mg, Se 0.3mg, V_A9000IU, V_{D3} 1600 IU, V_E 5 IU, V_K 0.5 mg, V_{B12} 0.004 mg, Biotin 0.1 mg, Folate 0.25 mg, Niacin 20 mg, Pantothenate 25 mg, V_{B6} 3 mg, V_{B2} 25 mg, Choline 500 mg.

Table S2 The primer sequences of the *aprA* gene and 16S rRNA gene

Gene[1]	Primer sequences
aprA	F: TGGCAGATMATGATYMACGG
	R: GGGCCGTAACCGTCCTTGA
16S rRNA	F: CGGCAACGAGCGCAACCC
	R: CCATTGTAGCACGTGTGTAGCC

[1] the target gene (*aprA*) and reference gene (16S rRNA).

Table S3 The production performance of laying hens [1]

Items	Lohmann	Hy-Line	*P*-value
Body weight, kg	1.77±0.02	1.75±0.02	0.605
ADFI, g	106.84±1.42	104.79±1.94	0.414
Laying rate, %	94.76±1.31	94.05±2.74	0.819
Average egg weight, g	59.34±0.64	58.86±0.50	0.567
Feed-egg ratio	1.89±0.04	1.97±0.04	0.173

[1] Data are presented as means with their standard errors.

Table S4 The recovery of respiration chamber system [1]

Chamber number	Recovery（%）	
	NH_3	H_2S
1	88.17±1.07	85.87±2.61
2	94.99±2.42	90.68±0.59
3	90.45±1.69	89.14±3.27
4	80.95±0.76	95.31±1.09
5	91.75±1.73	92.22±3.27
6	92.43±1.46	93.59±1.86
7	88.31±3.33	89.24±1.45
8	83.85±0.68	90.94±1.87
9	96.62±1.15	86.98±0.93
10	90.95±1.37	94.01±2.61
11	93.40±1.64	89.13±1.09
12	94.31±1.59	83.01±1.87

[1] Data are presented as means with their standard errors.

Table S5 Profile characteristics in cecal content and fermentation broth [1]

Items	Lohmann	Hy-Line	*P*-value
In vivo			
pH	6.68±0.15	6.70±0.17	0.929
Ammonium, mg/g	2.22±0.09	2.02±0.14	0.238
Sulfate radical, mg/g	2.27±0.17	2.07±0.16	0.398
In vitro			
pH	7.18±0.03	7.16±0.02	0.696
Ammonium, mg/mL	1.68±0.05[a]	1.52±0.03[b]	0.017
Sulfate radical, µg/mL	133.33±2.76[a]	113.36±2.32[b]	0.000

[1] Data are presented as means with their standard errors.
[a, b] Means within a row with different superscripts differ ($P < 0.05$).

Table S6 The relative abundance of the forty-two significantly different genera between two groups [1]

Genus	Hy-Line	Lohmann	*P*-value
Helicobacter	3.1236	1.2018	0.003
Parabacteroides	1.8391	0.5645	0.001
Sutterella	1.5792	2.4390	0.008
Alistipes	1.4977	2.8753	0.002
Lactobacillus	0.9246	1.5326	0.002
Butyricicoccus	0.4789	0.3229	0.035
Fournierella	0.4071	0.8056	0.002
Anaerofilum	0.2310	0.0621	0.001
Flavonifractor	0.2151	0.1081	0.001
Butyricimonas	0.1762	0.0625	0.001
Alloprevotella	0.1352	0.0414	0.018
Merdibacter	0.0887	0.0423	0.001
Sphaerochaeta	0.0479	0.0001	0.001
Unidentified Bacteria	0.0471	0.0179	0.019
Rikenella	0.0414	0.0081	0.003
Eisenbergiella	0.0400	0.0632	0.004
Mailhella	0.0356	0.0721	0.001
Aeriscardovia	0.0271	0.0388	0.050
Unidentified Mollicutes	0.0259	0.0524	0.013
Roseburia	0.0222	0.0069	0.001
Candidatus Arthromitus	0.0185	0.0085	0.012
Streptococcus	0.0123	0.0005	0.001
Unidentified Melainabacteria	0.0118	0.0028	0.034
Synergistes	0.0108	0.0260	0.001
Dielma	0.0105	0.0042	0.029
Anaerosporobacter	0.0052	0.0895	0.001
Chlamydia	0.0052	9.36E-05	0.001
Gallibacterium	0.0017	0.0066	0.012
Corynebacterium	0.0016	0	0.000
Candidatus Saccharimonas	0.0012	0.0058	0.001
Rothia	0.0008	9.36E-05	0.021
Capnocytophaga	0.0007	0	0.008
Anaerobiospirillum	0.0007	0	0.008
Lautropia	0.0006	0	0.031
Unidentified Victivallales	0.0005	0.0067	0.001
Lawsonia	0.0004	0.0571	0.003
Unidentified Burkholderiaceae	0	0.0057	0.001
Anaeroplasma	0	0.0268	0.001
Chujaibacter	0	0.0025	0.000
Unidentified Acidimicrobiia	0	0.0018	0.000

Bryobacter	0	0.0019	0.000
Dyella	0	0.0017	0.000
Arthrobacter	0	0.0007	0.008
Others	30.14	25.73	0.003

[1] Abundance was expressed in terms of % mean relative frequency.

Table S7 Correlation coefficients of some odor-related microbiota and odor production[1]

Genus	NH_3	H_2S
Desulfovibrio	-0.120	0.265*
Mailhella	-0.197	0.283*
Bilophila	-0.223	0.046
Lawsonia	-0.066	0.220*
Butyricicoccus	-0.055	-0.345**
Butyricimonas	-0.001	-0.390**
Roseburia	-0.144	-0.401**
Fusobacterium	0.293*	-0.097
Unidentified Clostridiales	0.361**	-0.076
Campylobacter	0.148	0.029

[1] NH_3 and H_2S production per kg average daily intake and the relative abundance of bacteria were applied for correlation analysis. ** means correlation is significant at 0.01 level; * means correlation is significant at 0.05 level.

Supplemental Figures:

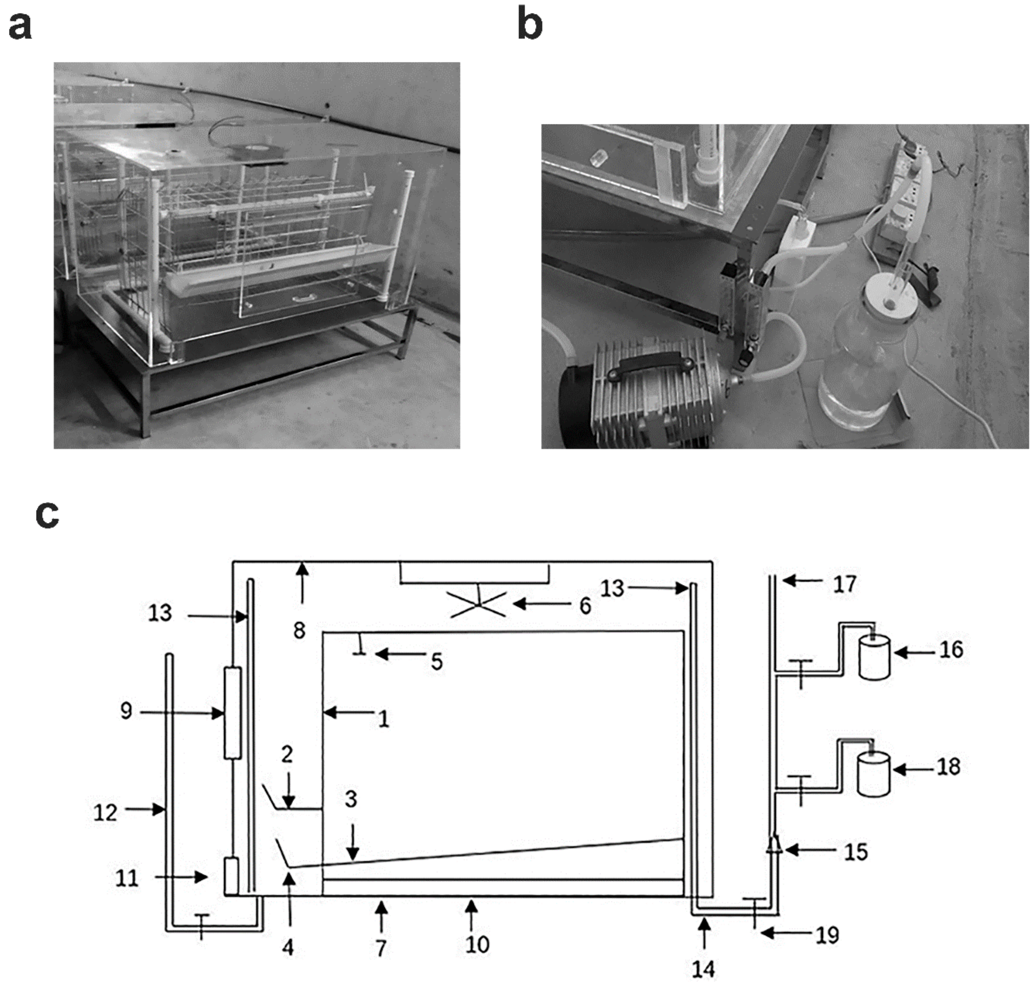

Fig. S1 The structure of respiration chamber system. **a** The physical picture of respiration chamber. **b** Absorption bottle for gas collection. **c** The sketch of respiration chamber. 1 The inner cage, 2 Feed trough, 3 Inner floor, 4 Eggs trough, 5 Drinker, 6 fan, 7 PVC floor, 8 Organic glass cover, 9 Baffle of feeding, 10 Feces collection, 11 Baffle of feces collection, 12 Air inlet, 13 Air outlet, 14 Total air outlet, 15 Air pump, 16 Ammonia absorption bottle, 17 Exhaust hose, 18 Hydrogen sulfide absorption bottle, 19 Flowmeter.

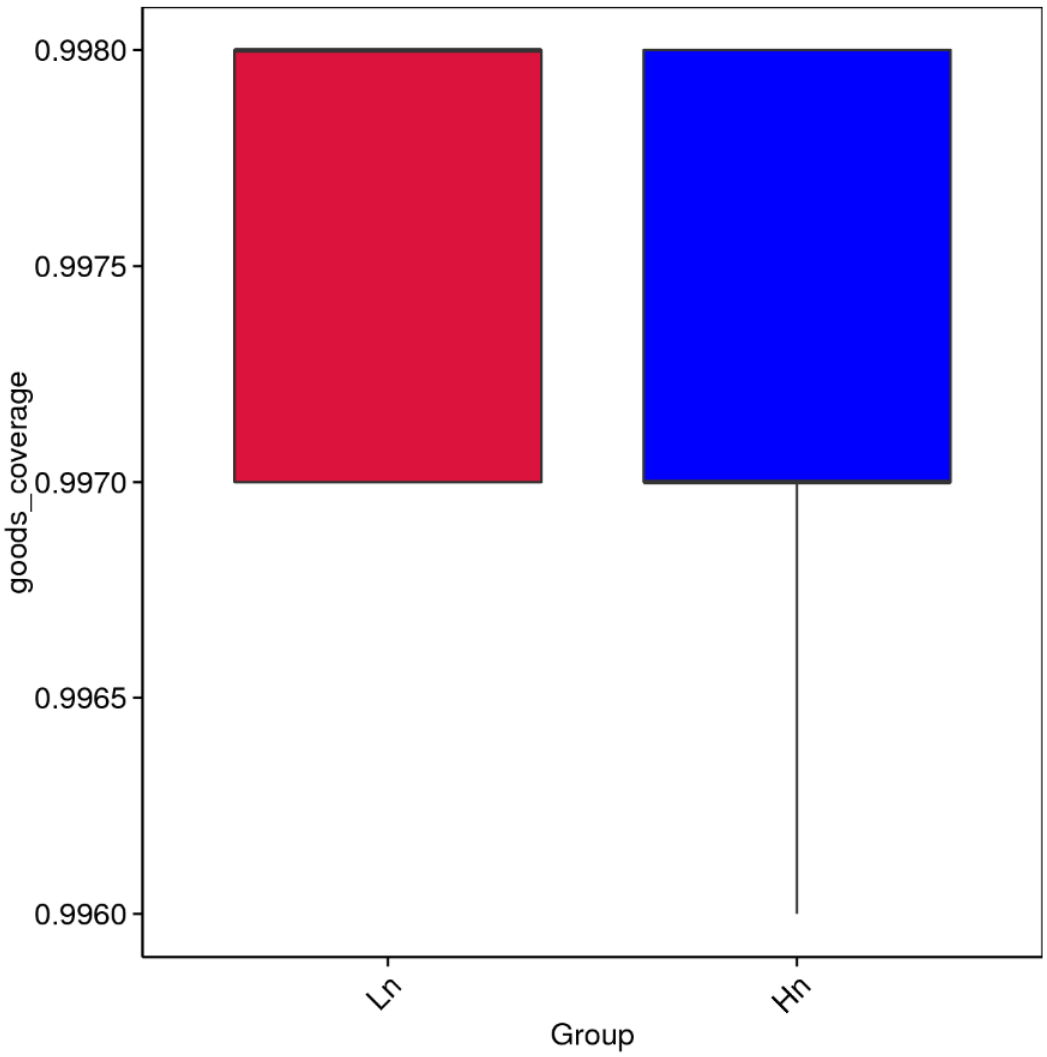

Fig. S2 The Good's coverage index box plots of two groups. Good's coverage index with a box plot exhibiting the depth of 16S rRNA sequencing. Hn means Hy-Line, and Ln means Lohmann.
